# T Helper 1 Cellular Immunity Toward Recoverin Is Enhanced in Patients With Active Autoimmune Retinopathy

**DOI:** 10.3389/fmed.2018.00249

**Published:** 2018-09-13

**Authors:** Steven K. Lundy, Enayat Nikoopour, Athanasios J. Karoukis, Ray Ohara, Mohammad I. Othman, Rebecca Tagett, K. Thiran Jayasundera, John R. Heckenlively

**Affiliations:** ^1^Department of Internal Medicine—Rheumatology, University of Michigan Medical School, Ann Arbor, MI, United States; ^2^Graduate Training Program in Immunology, University of Michigan Medical School, Ann Arbor, MI, United States; ^3^Department of Ophthalmology and Visual Sciences—Kellogg Eye Center, University of Michigan Medical School, Ann Arbor, MI, United States; ^4^Biostatistics Core Facility, University of Michigan Medical School, Ann Arbor, MI, United States

**Keywords:** retinal diseases, autoimmunity, recoverin, cancer immunotherapy, cell mediated immunity, interleukin 10, interferon-gamma, Nanostring technologies

## Abstract

Autoimmune retinopathy (AIR) causes rapidly progressive vision loss that is treatable but often is confused with other forms of retinal degeneration including retinitis pigmentosa (RP). Measurement of anti-retinal antibodies (ARA) by Western blot is a commonly used laboratory assay that supports the diagnosis yet does not reflect current disease activity. To search for better diagnostic indicators, this study was designed to compare immune biomarkers and responses toward the retinal protein, recoverin, between newly diagnosed AIR patients, slow progressing RP patients and healthy controls. All individuals had measurable anti-recoverin IgG and IgM antibodies by ELISA regardless of disease status or Western blot results. Many AIR patients had elevated anti-recoverin IgG1 levels and a strong cellular response toward recoverin dominated by IFNγ. RP patients and controls responded to recoverin with a lower IFNγ response that was balanced by IL-10 production. Both AIR and RP patients displayed lower levels of total peripheral blood mononuclear cells that were due to reductions of CD4^+^ T_H_ cells. A comparison of messenger RNA (mRNA) for immune-related genes in whole blood of AIR patients versus RP patients or controls indicated lower expression of ATG5 and PTPN22 and higher expression of several genes involved in T_H_ cell signaling/transcription and adhesion. These data indicate that an immune response toward recoverin is normal in humans, but that in AIR patients the balance shifts dramatically toward higher IFNγ production and cellular activation.

## Introduction

Autoimmune retinopathy (AIR) is a retinal degenerative disease that results in an acute and rapid loss of peripheral vision, night blindness and other visual defects. The original discovery of AIR resulted from analysis of a number of patients referred for retinitis pigmentosa (RP) who did not have the classical presentation, but instead had retinas that looked normal or were hypo-pigmented (1, 2). These patients often did not have family histories of genetic polymorphisms commonly associated with RP, but instead many had significant personal or family histories of autoimmunity, or a comorbidity of melanoma or another type of cancer. The rapid progression and other distinguishing clinical characteristics in these patients led to the hypothesis that it was the result of immune pathogenic mechanisms rather than retinal degeneration caused by RP-associated mutations (3–5). To determine if autoimmunity against retinal antigens was present in these patients, Western blot and histological assays have been developed to detect anti-retinal antibodies (ARA) as laboratory diagnostic tests (6–8). Patients with either the non-paraneoplastic (npAIR) or cancer-associated forms of AIR (CAR) often tested positive for anti-retinal IgG antibodies with specificities toward more than one retinal protein (9, 10). This is in contrast with RP patients or healthy control subjects who sometimes have weak reactivity toward one to three retinal proteins or test negative on the ARA Western blot (11). The importance of a correct diagnosis is highlighted by the fact that many AIR patients experienced stabilization or improvement of their visual fields following general immunosuppressive treatments (12). Thus, it is critically important to develop methods for early detection of AIR, and to have better defined treatments for AIR in order to preserve vision in these patients.

Although ARA are highly supportive of the diagnosis of AIR, they do not have a well-defined role in AIR pathogenesis (13). It has been suggested based on *in vitro* studies and *in vivo* studies in rats that binding of ARA to retinal cells induces programmed cell death (apoptosis), however this is not proven to happen *in vivo* in humans (14, 15). The sizes of proteins recognized by ARA on Western blots vary widely between individuals, with no clearly dominant specificities being found in a majority of patients (10). Among the retinal antigens that have been identified to date, one of the most common is recoverin, a 23 kilodalton calcium binding photoreceptor protein that functions as an inhibitor of rhodopsin kinase and regulates the recovery phase of light detection (16, 17). Recoverin is normally a cytosolic protein that when activated by binding of calcium attaches to the intra-cellular disc membrane of the photoreceptor cell. It is not commonly thought to associate with the outer plasma membrane, where it could be recognized by circulating antibodies that would mediate apoptosis. This localization makes it unlikely that the anti-recoverin antibodies found in some AIR patients, and that are especially prevalent in patients with CAR, play a direct pathogenic role in retinal attack. Instead, we hypothesized that poorly regulated cellular immunity toward recoverin might be a more important trigger of AIR pathogenesis that can secondarily lead to autoantibody production (18).

In the current study, we compared the anti-recoverin immune responses of healthy individuals, RP patients with no indication of autoimmunity, and newly-diagnosed npAIR patients whom had not yet received immune suppressive treatments. As expected, npAIR patients had significantly high numbers of anti-retinal antibodies on Western blots with seemingly random specificities. However, a more sensitive ELISA technique showed that most individuals had measurable titers of anti-recoverin IgG and IgM antibodies regardless of disease status, and only anti-recoverin IgG1 titers were significantly higher among a subset of AIR patients. Peripheral blood cells from both patient groups and control subjects had a measurable reaction toward recoverin that was dominated by the anti-inflammatory cytokine, interleukin-10 (IL-10), suggesting a natural and systemic immune tolerance was present toward this retinal antigen. In contrast, cells from npAIR patients also responded to recoverin with elevated levels of the T_H_1-type, proinflammatory cytokine, interferon gamma (IFNγ). A comparison of whole blood mRNA between these groups revealed some differences in gene expression that may be linked to disease pathogenesis. These data are the first to directly demonstrate that recoverin may act as a normal tolerogenic antigen in humans, and that a T_H_1-mediated immune response toward recoverin is a common feature among newly diagnosed AIR patients. The potential implications of these findings to understanding the pathogenesis, and improving diagnosis and treatment of CAR and npAIR are discussed.

## Materials and methods

### Patient and control blood samples

All autoimmune retinopathy (AIR) and retinitis pigmentosa (RP) patients were evaluated in the Retinal Dystrophy Clinic of the Kellogg Eye Center at the University of Michigan. This study was carried out in accordance with the recommendations of the Policy for Protection of Human Research Subjects published by the U.S. Department of Health and Human Services. The protocol was approved by the University of Michigan Institutional Review Board. All subjects signed written informed consent in accordance with the Declaration of Helsinki. AIR diagnosis was based on clinical presentation consistent with previously reported parameters (6, 19). These include: late onset, rapid and significant loss of peripheral vision, decreased electro-retinogram (ERG) responses, and normal or hypo-pigmented retina as detected by fundus examination. AIR patients in this study ranged in age between 29 and 71 years when diagnosed and only were eligible if they were not previously diagnosed with any malignancy and had not received immune suppressive treatments within the previous 90 days. Gender, age, and race were not part of the exclusion criteria for study participation. RP patients had classical presentations of slower vision loss and pigment deposits on their retinas. Peripheral blood was obtained from a total of 15 AIR and 15 RP patients, and from 14 healthy controls with no reported visual defects. Peripheral blood was collected in vacutainer tubes (BD Biosciences, Franklin Lakes, NJ) following standard procedures and processed within 24 h under sterile conditions.

### Anti-retinal antibody western blot

Patient and control serum was collected in 5 mL BD Vacutainer SST^TM^ tubes and was separated from clotted blood after 30 min incubation at room temperature by centrifugation at 1200 × g for 15 min. Aliquoted serum was stored at 80°C below zero until the day of assay. Retinal tissue was carefully dissected from human organ donors with normal vision at the time of death (Midwest Eye Bank/Eversight, Ann Arbor MI) and lightly sonicated in 5 mL of TBS buffer containing proteinase inhibitors. Proteins extracted from the retinal homogenate were stored for later use at −80°C in 50 μg aliquots. Upon thawing, the retinal proteins were heated at +80°C for 10 min and then 30 μg of protein was separated on a 10% NuPAGE gel (Life Technologies, Grand Island, NY) and run next to a SeeBlue Plus2 molecular weight standard (Life Technologies) at 100 V for 1 h in MOPS buffer. Separated proteins were transferred to nitrocellulose membrane using standard Western blot techniques and washed 3 times with MOPS buffer prior to blocking with 3% milk/1 × PBS/0.2% Tween20 solution for 1 h. Patient serum was thawed and diluted 1:100 in 1 × PBS and added to the blot overnight at 20°C. After 3 washes with MOPS buffer, binding of patient antibodies was detected with a 1:10,000 dilution of goat anti-human IgG (H + L) alkaline phosphatase conjugated antibodies (BD Pharmingen, San Diego, CA) followed by addition of Nitro Blue Tetrazolium and 5-Bromo-4-chloro-3-indolyl phosphate (Sigma Aldrich, St. Louis, MO) and exposure for 10 min before stopping the reaction by rinsing with water.

### Anti-recoverin antibody enzyme-linked immunosorbent assays

Patient and control plasma was separated from heparinized peripheral blood by centrifugation at 900 × g for 10 min and aliquots were stored at −80°C for later analysis. Recombinant human recoverin (MyBioSource, San Diego, CA) was diluted to 2 μg/mL in pH 9.5 Carbonate buffer and 0.1 mL was added to triplicate wells of EIA plates for each patient and antibody isotype tested. Sets of triplicate wells/patient/isotype were set up with 0.1 mL of Carbonate buffer without protein as no antigen controls and the plate was incubated overnight at +4°C to allow binding of the human recoverin protein. All wells were washed 3× with ELISA wash buffer (1 × PBS/0.2%Tween20) followed by addition of 0.25 mL of blocking buffer (1 × PBS/2% bovine serum albumin) and incubation for 1 h in the dark at room temperature. After blocking, the buffer was removed and wells were washed 3× with ELISA wash buffer and blotted on paper towels to remove excess liquid. Patient or control plasma was diluted 1:40 in 1 × PBS and 0.1 mL was added to each of the triplicate wells coated with recoverin or left uncoated per antibody isotype. Plates were incubated for an additional 2 h at room temperature. Diluted plasma was removed, and plates were washed 3× with ELISA wash buffer before addition of 0.1 mL of 1:200 dilutions in blocking buffer of either biotinylated anti-human IgG or biotinylated anti-human IgM (BD Pharmingen) followed by 1 h incubation at room temperature. For anti-recoverin IgG1 detection, the same protocol was followed except that a 1:2,000 dilution of biotinylated anti-human IgG1 antibody (BD Pharmingen) was used for detection. After an additional 5 washes, 0.1 mL of streptavidin-horse radish peroxidase (SA-HRP) reagent was added to each well and incubated for 30 min at room temperature. Unbound SA-HRP was removed by 7 washes with 0.2 mL ELISA wash buffer, and bound reagent was detected by color change of TMB Substrate (BD Biosciences) during a 4–10 min incubation that was stopped by addition of 2N H_2_SO_4_ buffer. Absorbance of 450 nm light was analyzed using Epoch 2 spectrophotometer and Gen 5 software (BioTek Instruments, Winooski, VT).

### Anti-human recoverin cytokine response

Peripheral blood mononuclear cells (PBMC) were separated from 15–25 mL of heparinized blood by density gradient centrifugation on Ficoll-Histopaque^TM^ (Sigma Aldrich, St. Louis, MO). Viable PBMC (>95% of total in all samples) were counted with a hemocytometer using trypan blue dye exclusion. Total cell numbers reported in the figures and used to establish cell cultures were determined based on live cell counts. Purified PBMC (0.2 mL/well) were placed into 96 well round bottom plates at a concentration of 1-2 million cells/mL in culture medium consisting of 1× RPMI supplemented with 10% heat-inactivated fetal calf serum, 4 mM L-glutamine, 100 U/mL penicillin, and 100 μg/mL streptomycin (Gibco, Grand Island, NY). Triplicate wells were pulsed with 2.5 μg/mL recombinant human recoverin (MyBioSource), 10 ng/mL staphylococcal enterotoxin A (Toxin Technologies, Sarasota, FL) as a positive control, or cell culture medium only as a negative control. Prior to use in these assays, the commercial recombinant recoverin tested negative for lipopolysaccharide contamination by limulus amoebocyte assay. Cultures were incubated at 37°C, 95%, humidity and 7.5% carbon dioxide for 6 days without medium change or additional stimulation at which time cell-free supernatants were collected and stored at −80°C prior to cytokine measurement by ELISA using kits and manufacturer's protocols. The concentrations of human interferon gamma (IFNγ) and interleukin-10 (IL-10) in the culture supernatants were determined against a standard curve supplied with the corresponding OptEIA kit (BD Biosciences, San Jose, CA). Tumor necrosis factor alpha (TNFα) concentrations were determined using DuoSet kits purchased from R & D Systems (Minneapolis, MN).

### Flow cytometry

Isolated PBMC were stained with monoclonal antibodies reactive against human cell surface markers of major lymphocyte lineages and T cell subsets. To determine the distribution of major cell lineages in patient PBMC, the following fluorochrome-conjugated antibodies were used: CD3-PE/Cy7 (Clone: SK7), CD8-FITC (Clone: G42-8), CD19-V500 (Clone: HIB19), and CD56-PerCP/Cy5.5 (Clone: B159) from BD Pharmingen; and CD4-APC (Clone: RPA-T4) from BioLegend (San Diego, CA). In a separate staining panel naïve and memory T cell distribution was determined using BioLegend CD45RA-Pacific Blue (Clone: HI100) and CD45RO-Alexa700 (Clone: UCHL1) antibodies, respectively, in combination with the CD3, CD4 and CD8 antibodies described above. CCR7-PerCP/Cy5.5 antibody (Clone: G043H7, BioLegend) binding was used to distinguish central and effector memory subsets in the memory T cell panel. Staining was detected using an LSR II flow cytometer (BD Biosciences) in the Vision Core of the Kellogg Eye Center. Flow cytometric data was analyzed using FlowJo version X.0.6 software (TreeStar, Ashland, OR) using the gating strategies shown in the Supplementary Figures 2, 3. Cell gating was determined by comparing stained cells with unstained PBMC and confirmed using staining with all but one of the antibodies in each panel. Lymphocyte gating based on forward scatter versus side scatter, and singlet gating using forward scatter height versus forward scatter area was performed on all samples prior to analysis of cell surface markers. Percentages of monocytes and granulocytes were determined by forward scatter and side scatter characteristics using the gating strategy shown in Supplementary Figure 2.

### RNA isolation and nanostring analysis

RNA was purified from whole blood collected in PAXgene^TM^ Blood RNA tubes (BD Biosciences) using PreAnalytiX (Qiagen GmbH, Germany) PAXgene RNA isolation kits and manufacturer's protocol. The concentration of mRNA was determined using a NanoDrop 1000 (ThermoFisher Scientific) apparatus, and quality was confirmed by running 1 μL in Bioanalyzer 2100 (Agilent Technologies, Santa Clara, CA). A 400 ng aliquot of purified RNA was sent to the NanoString Research Support Facility at Michigan State University (East Lansing, MI) where 100 ng of RNA was mixed with the nCounter^TM^ Human Immunology Panel (NanoString Technologies, Seattle, WA). The Human Immunology panel measures over 700 genes that include cytokines, chemokines, transcription factors, signaling molecules and receptors involved in various types of immune responses. Binding to mRNA was detected with a NanoString nCounter Analysis System and the raw count data was normalized and statistically analyzed as described below by the Bioinformatics Core of the University of Michigan. The normalized mRNA copy number data from this study has been deposited in the NIH GEO database at: Accession #GSE117751 https://www.ncbi.nlm.nih.gov/geo/query/acc.cgi?acc=GSE117751.

### Data collection and statistical analysis

ELISA for anti-recoverin antibodies and culture-derived cytokines were done in triplicate for all samples. Dots on the graphs produced using Prism 6.01 (GraphPad Software Inc., La Jolla, CA) represent the mean value for each individual. The median and 95% confidence intervals for each group are indicated with lines and hash marks on the graphs. Significant differences between the three patient groups were determined by Kruskall-Wallis tests, and the exact *P*-values for each group to group comparison shown below the titles of the figures were determined by Mann-Whitney *t*-tests. *P*-values <0.05 were considered to be significant. Flow cytometric data was obtained from 20,000 or more cells within the lymphocyte gate for each subject. Gating shown in the supplementary figures was found to be consistent throughout the study and was used for all samples. Statistics for flow cytometry data were performed using the Prism 6.01 program. Data shown are median ± 95% confidence intervals for each group. Stacking bar graph representations of the flow cytometry data were created using Excel software (Microsoft Inc., Seattle, WA). Raw mRNA copy number data from NanoString analysis was filtered to include mRNA expression above 32 copies/100 ng total RNA and then data was linearized and Voom normalized using the R statistics package. A significant batch effect was detected between samples analyzed before and those run in 2017. The batch effects were corrected using the Combat method, and confirmed by analyzing the relative levels of spike-in control mRNA and several housekeeping genes as shown in Supplementary Figures 4, 5.

## Results

### Patient identification and ARA western blot analysis

**Figure 1:**
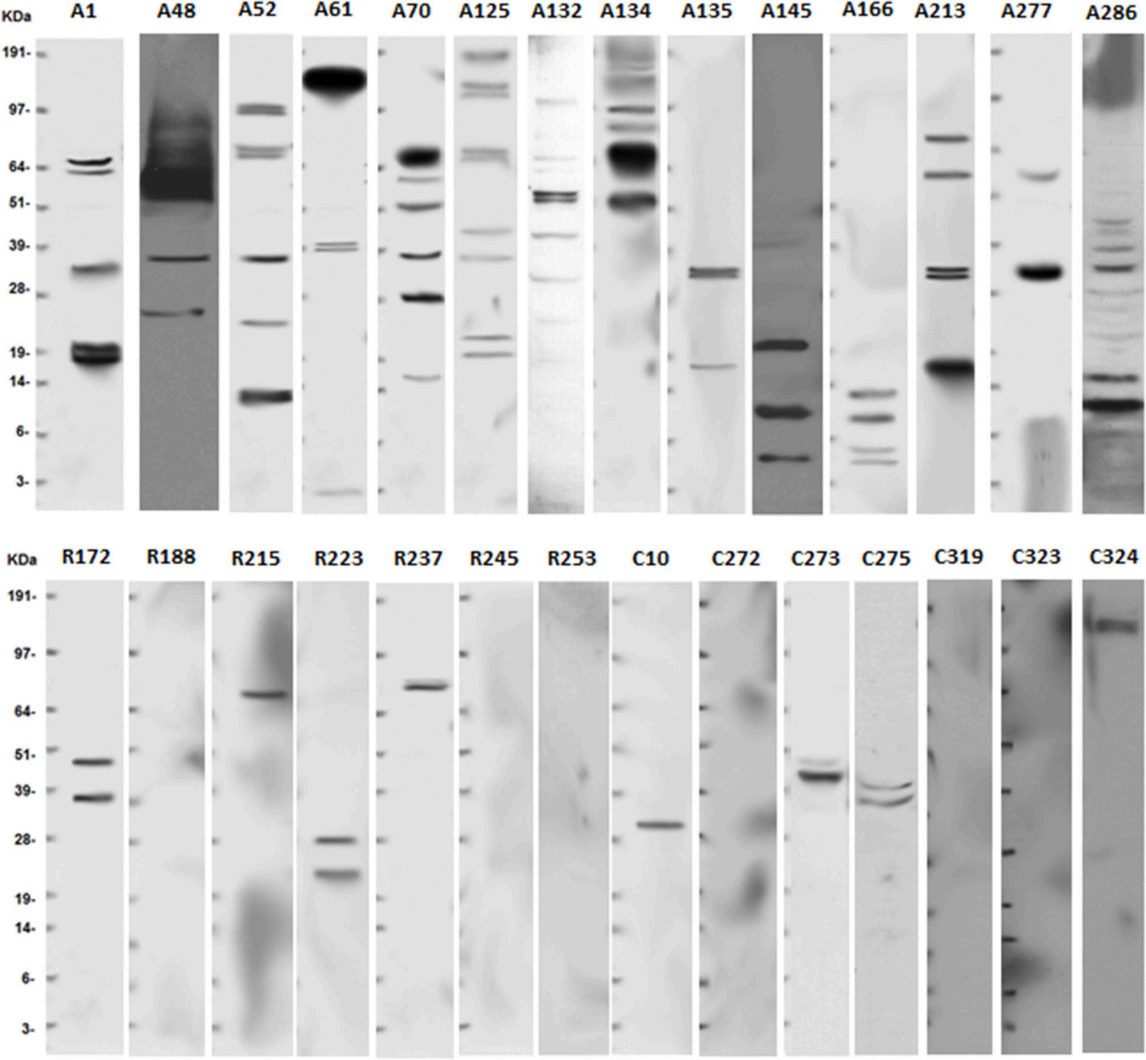
Anti-retinal antibodies are more common in serum of AIR patients than in non-autoimmune RP patients or controls. Serum was collected from newly diagnosed and untreated AIR (A#) patients, RP (R#) patients, or healthy control (C#) individuals. Human retinas were isolated from cadaver eyes provided by the Michigan Eye Bank/Eversight. Retinal proteins were prepared by homogenization and 30 μg of protein was separated on a 10% NuPAGE gel by using standard electrophoresis methods. Proteins were transferred to a nitrocellulose membrane that was used to capture anti-retinal antibodies from a 1:100 dilution of patient serum. Binding was detected using biotinylated anti-human IgG antibody, horseradish peroxidase-conjugated streptavidin, and SuperSignal West Pico Chemiluminescent Substrate. Exposure to auto-radiography film was for 10 minutes, and SeeBlue Plus 2 molecular weight standard was run on each gel to align protein sizes. Representative blots are shown for RP patients and controls, but all individuals were tested.

Following the informed consent on this IRB approved protocol, peripheral blood samples were collected from 15 npAIR patients, 15 RP control patients and 14 healthy controls. Demographics of the patients are summarized in Table 1 and individual demographic information is found in Supplementary Table 1. The AIR patients were all newly diagnosed and untreated with no reported family histories of RP or other retinal degeneration, and no personal histories of cancer. None of the AIR patients in this study has subsequently been diagnosed with RP or cancer. The majority of the RP control patients were on long-term follow up with slowly progressing vision loss, with the remainder being newer patients with family histories and/or classic symptoms of RP and no personal histories of autoimmunity or cancer. A group of healthy individuals with no reported vision loss, autoimmune diseases or cancers was recruited to closely match the age, and gender demographics of the AIR patients (Table 1 and Supplementary Table 1). Detection of ARA using Western blots (Figure 1) showed the expected pattern of high activity among the AIR patients and low to no ARA bands for the controls or RP patients.

**Table 1:** Grouped patient demographics.

**Patient category**	**Gender**	**Number (% within group)**	**Mean Age + StDev (years)**	**Age range (years)**
Autoimmune retinopathy	Female Male	10 (66.7%) 5 (33.3%)	55.8 + 12.1 56.0 + 15.9	40–71 29–71
Retinitis pigmentosa	Female Male	9 (60.0%) 6 (40.0%)	48.7 + 15.4 46.3 + 22.8	20–67 20–74
No eye disease controls	Female Male	9 (64.3%) 5 (35.7%)	56.2 + 8.4 57.2 + 8.6	39–69 50–68

### All individuals have anti-recoverin antibodies

**Figure 2:**
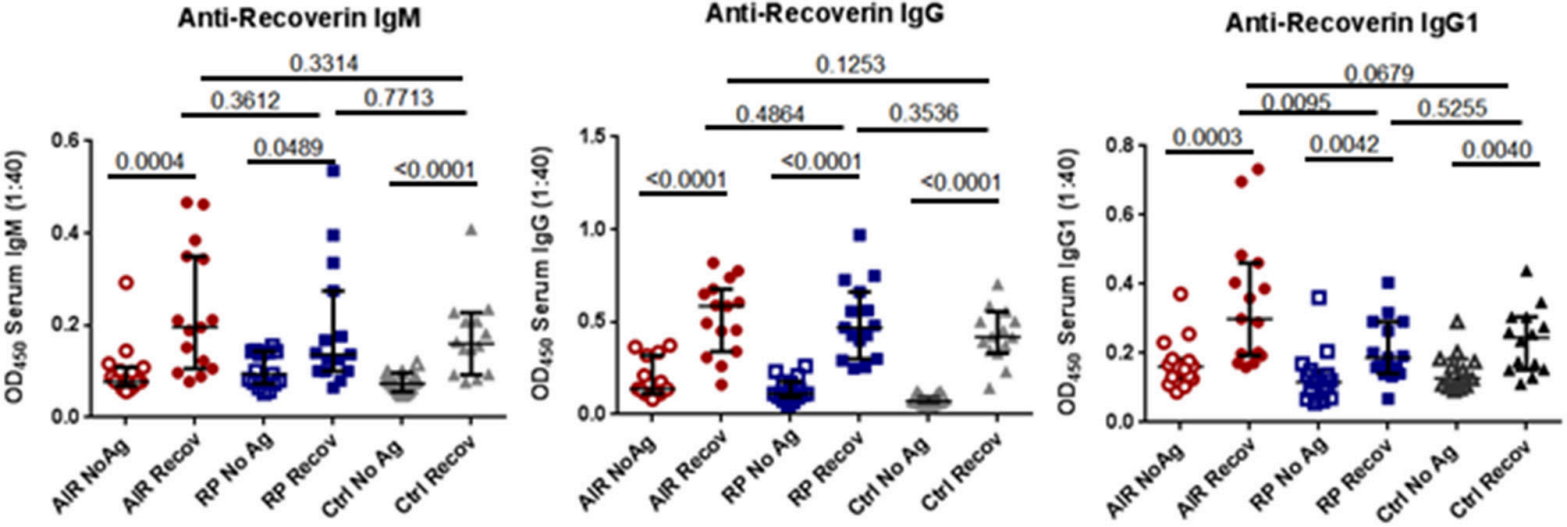
Anti-recoverin antibodies are frequently present in blood of patients and controls. Plasma was separated from heparinized blood of untreated AIR patients (*n* = 15), RP patients (*n* = 15), and healthy controls (*n* = 14). Half of the wells of a flat-bottomed 96 well EIA plate were coated overnight with 2.5 μg/mL recombinant human recoverin (Recov) in carbonate buffer, washed, and blocked with PBS 2% BSA for 2 h. Control wells were similarly treated without addition of recoverin (NoAg). A 1:40 dilution of plasma was added in triplicate to Recov and NoAg wells for each sample for 2 h. Plates were then washed and binding of patient antibodies was detected in replicate wells using biotin-conjugated anti-human IgM-, anti-human IgG-, or anti-human IgG1-specific antibodies, followed by addition of streptavidin-HRP and TMB substrate. Each dot represents the mean 450 nm absorbance for one individual run in triplicate. Black lines and hash marks show the median and 95% confidence interval for each group and the exact *P*-values as determined by Mann-Whitney tests are shown for the indicated pairwise comparisons.

In order to have a more sensitive and specific measurement of anti-recoverin antibodies, we developed an ELISA (detailed description in Methods) using plates coated with recombinant human recoverin to capture antibodies from human plasma. As shown in Figure 2, nearly all of the individuals in the study, including the RP patients and healthy controls, had a measurable amount of anti-recoverin antibody binding above the background non-specific binding to the plates. Both IgG and IgM anti-recoverin antibodies were detectable in most individuals, however, median levels of anti-recoverin IgG1 were significantly higher in the AIR patients compared to RP patients (*P* = 0.0095). Despite several attempts to quantify the other IgG isotypes, the overwhelming majority of samples were below detection limits of the assays (data not shown). The numerical mean OD_450_ values ± standard deviations of triplicate wells for total anti-recoverin IgM, IgG, and IgG1 for each individual are presented in Supplementary Table 1.

### T cell responses toward recoverin differ between AIR and other groups

**Figure 3:**
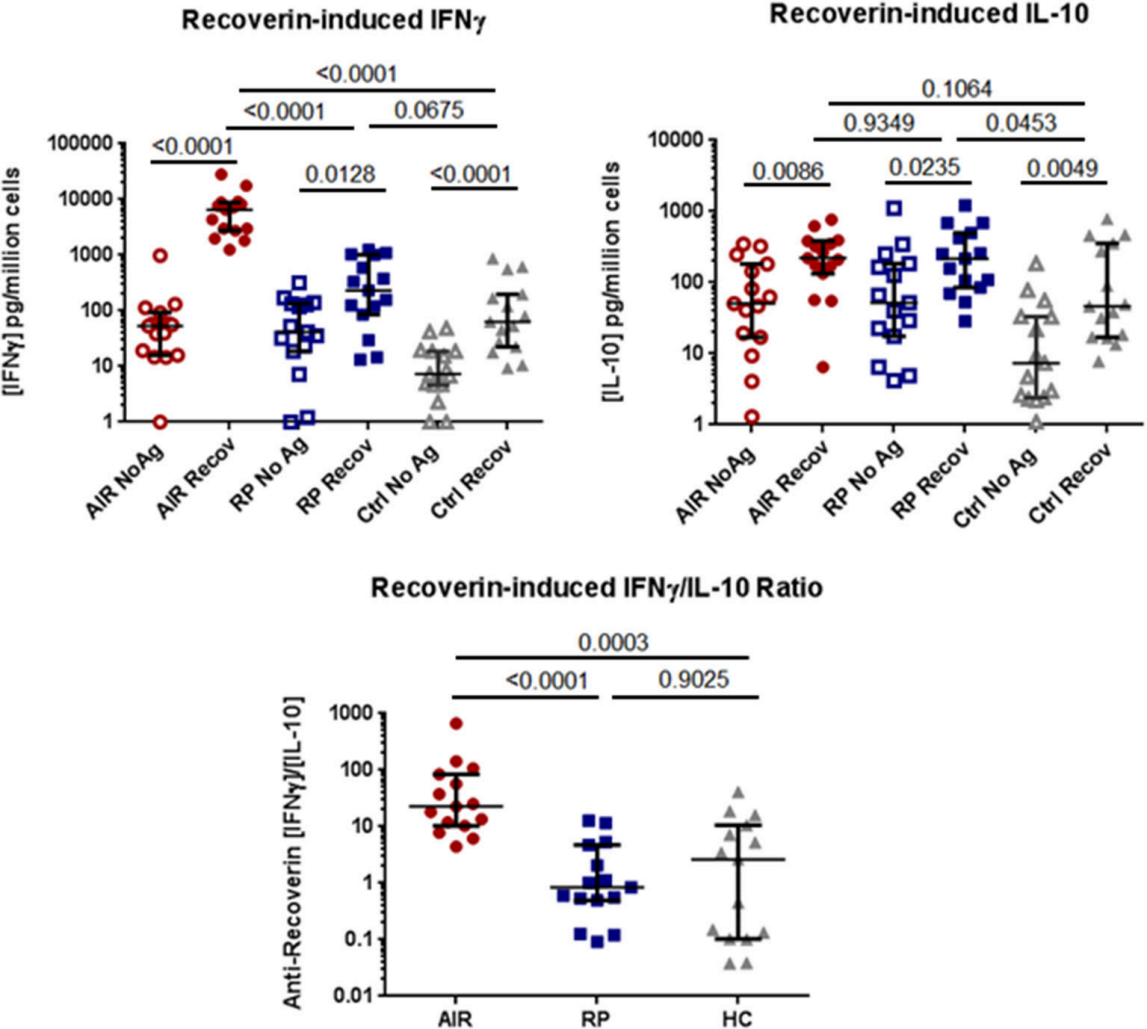
Comparison of PBMC cytokine production in response to recoverin. Isolated PBMC from AIR patients (*n* = 15), RP patients (*n* = 15) and healthy controls (*n* = 14) were cultured for six days in the presence or absence of 2.5 μg/mL of recombinant human recoverin. Culture supernatants were collected and the levels of released IFNγ and IL-10 were determined by commercial sandwich ELISA. Each dot represents the mean cytokine level from triplicate wells for each individual. The group median and 95% confidence intervals are shown with horizontal lines and hash marks, respectively. Exact *P-*values determined by non-parametric Mann-Whitney tests are expressed below the title of each graph. The ratio of IFNγ to IL-10 production for each individual is shown on the lower graph.

The results from the anti-recoverin antibody ELISA suggested that immune responses toward recoverin were much more common than previously reported. To determine whether T helper (T_H_) cell responses toward recoverin were measurable in these individuals, cell culture assays were performed. PBMC were isolated from the same cohort of untreated AIR patients (*n* = 15), RP patients (*n* = 15), and healthy controls (*n* = 14) and cultured in the presence or absence of recombinant human recoverin. The release of cytokines IFNγ and IL-10 were analyzed, and as shown in Figure 3, every participant had a measurable response following incubation with recoverin for at least one cytokine.

Notably, the AIR patients had consistently and significantly higher production of IFNγ in response to recoverin than either RP patients (*P* < 0.001) or controls (*P* < 0.001), whose PBMC were similarly activated to produce this T_H_1-associated cytokine. In contrast, the release of the immune regulatory cytokine IL-10 in response to recoverin was not significantly different between all three groups. Analysis of the ratio of IFNγ/IL-10 for each individual revealed that all of the AIR patients had a higher ratio than the median value for the RP patients or healthy controls. PBMC from all of these patients and controls released IFNγ in response to superantigen staphylococcal enterotoxin A (SEA), a positive control (Supplementary Figure 1A).

Prior to this study, production of IL-17 by PBMC in response to recoverin was measured for a large number (*n* > 50) of treated and untreated AIR patients as well as RP patients and controls. IL-17 production was negative in response to recoverin in the vast majority of people (data not shown). Tumor necrosis factor alpha (TNFα) production is another marker of T_H_1 activation. In the 11 of 15 AIR patients in this cohort who were tested for production of TNFα in response to recoverin, several had elevated TNFα responses toward recoverin compared to people in the two other cohorts (Supplementary Figure 1B).

### CD4^+^ T_H_ cell levels are lower in AIR and RP patients

**Figure 4:**
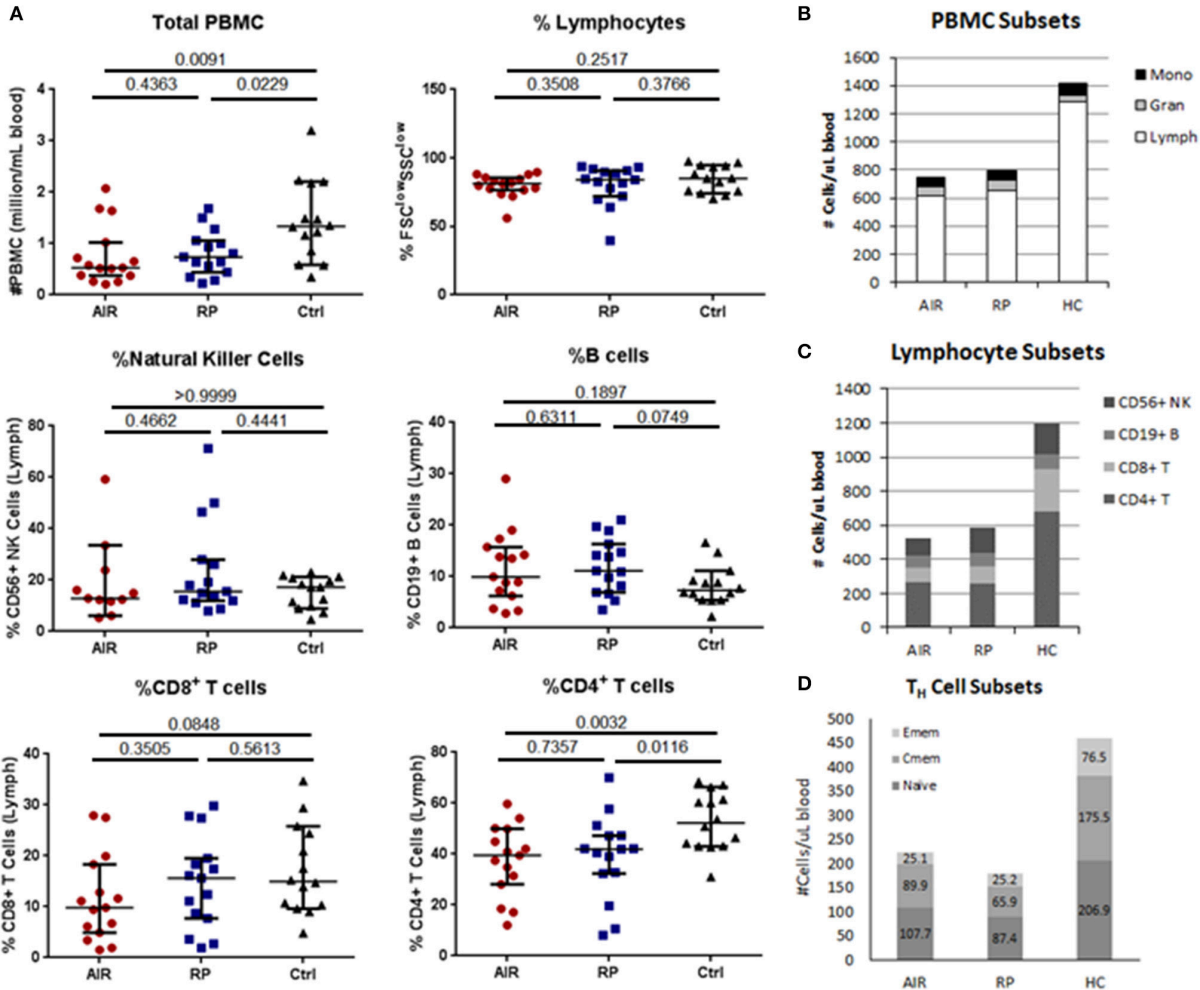
Comparison of major lymphocyte subsets in blood of AIR or RP patients and controls. Isolated PBMC from 15 AIR patients, 15 RP patients and 14 healthy controls were counted and stained with fluorochrome-conjugated antibodies specific for human CD3, CD4, CD8, CD56, and CD19 **(A–C)**. **(A)** Peripheral blood mononuclear cells were counted using trypan blue and then stained for flow cytometry. Lymphocytes were gated as small, non-granular cells using forward scatter and side scatter. Percentages of CD56^+^CD3^neg^ natural killer cells, CD19^+^CD3^neg^ B cells, CD8^+^CD3^+^ cytotoxic T cells, and CD4^+^CD3^+^ T helper cells within the lymphocyte gate were determined for each individual. Horizontal lines mark the median and 95% confidence intervals for each group. Exact *P*-values from Mann-Whitney tests are shown below the title of each graph. **(B)** The percentages of lymphocytes, monocytes and granulocytes were determined using forward scatter and side scatter and multiplied by total PBMC counts to determine the number of each cell type per microliter of blood. Stacking bars represent the mean for each patient group. **(C)** Bar graph of average numbers of lymphocytes in each subset which were determined by multiplying the percentages and total lymphocyte counts for each sample. **(D)** A separate staining panel consisting of antibodies against CD3, CD4, CD8, CD45RA, CD45RO, and CCR7 was used to find percentages of naïve, central memory and effector memory T helper cell subsets (see Materials and Methods for criteria) which were multiplied by total numbers of CD4^+^CD3^+^ T_H_ cells in each sample.

To determine if the increased IFNγ response toward recoverin in AIR patients resulted in changes to lymphocyte subsets, we measured the relative proportions of circulating lymphocyte subsets between the untreated AIR patients, RP patients and healthy controls by flow cytometry. Percentages of major lymphocyte subsets (Figures 4A–C) and naïve and memory T cell subsets (Figure 4D) were quantified (see Supplementary Figures 2, 3 for gating) and multiplied by total PBMC counts. Significantly lower total numbers of PBMC were found in the blood of most of the AIR and RP patients when compared to the average for healthy controls (Figure 4A). Analysis of forward scatter and side scatter indicated similar percentages of lymphocytes (Figure 4A) in the three patient groups. Among the lymphocytes, the percentages of CD3^neg^CD56^+^ natural killer (NK) cells, CD3^neg^CD19^+^ B lymphocytes, and CD3^+^CD8^+^ CTL were similar between all groups, and only the CD3^+^CD4^+^ T_H_ lymphocyte subset showed a relative deficit in both the AIR and RP patients compared to healthy controls (Figure 4A). The decrease in total PBMC was solely attributable to low lymphocyte numbers since similar amounts of monocytes and granulocytes were found in all three groups (Figure 4B). The combination of low total PBMC number and low percentage of T_H_ cells resulted in extremely low total numbers of circulating T_H_ cells in both patient groups (Figure 4C). An analysis of naïve, central memory and effector memory T_H_ cell subsets showed that the decrease in both patient groups happened in all three T_H_ subgroups but was most profound in the effector memory subset where 2/3 of the cells were absent compared to the healthy control group (Figure 4D).

### AIR patients have significant changes in immune mediator gene expression

To analyze whether there were systemic differences in immune-associated gene expression, mRNA levels in whole blood of a set of immunologically important genes were quantified from 14 people in each group using the NanoString Human Immunology Panel. Expression of the housekeeping genes GAPDH, OAZ1, TUBB, and TBP were similar between all three groups (Supplementary Figure 5A). Gene expression of the major lymphocyte markers CD8, CD56, and CD19 was similar between the three groups but expression of the CD4 gene was higher in the AIR patients than in RP patients or controls (Supplementary Figure 5B). Of the 445 immune-associated genes analyzed, 86 genes were differentially expressed with an adjusted *P*-value below 0.05, and 42 genes had a *P*-value below 0.025 between the healthy controls and AIR patients. A listing of the 42 most significantly different genes is included in Supplementary Table 2 and the full data set has been posted to the NIH GEO database.

**Figure 5:**
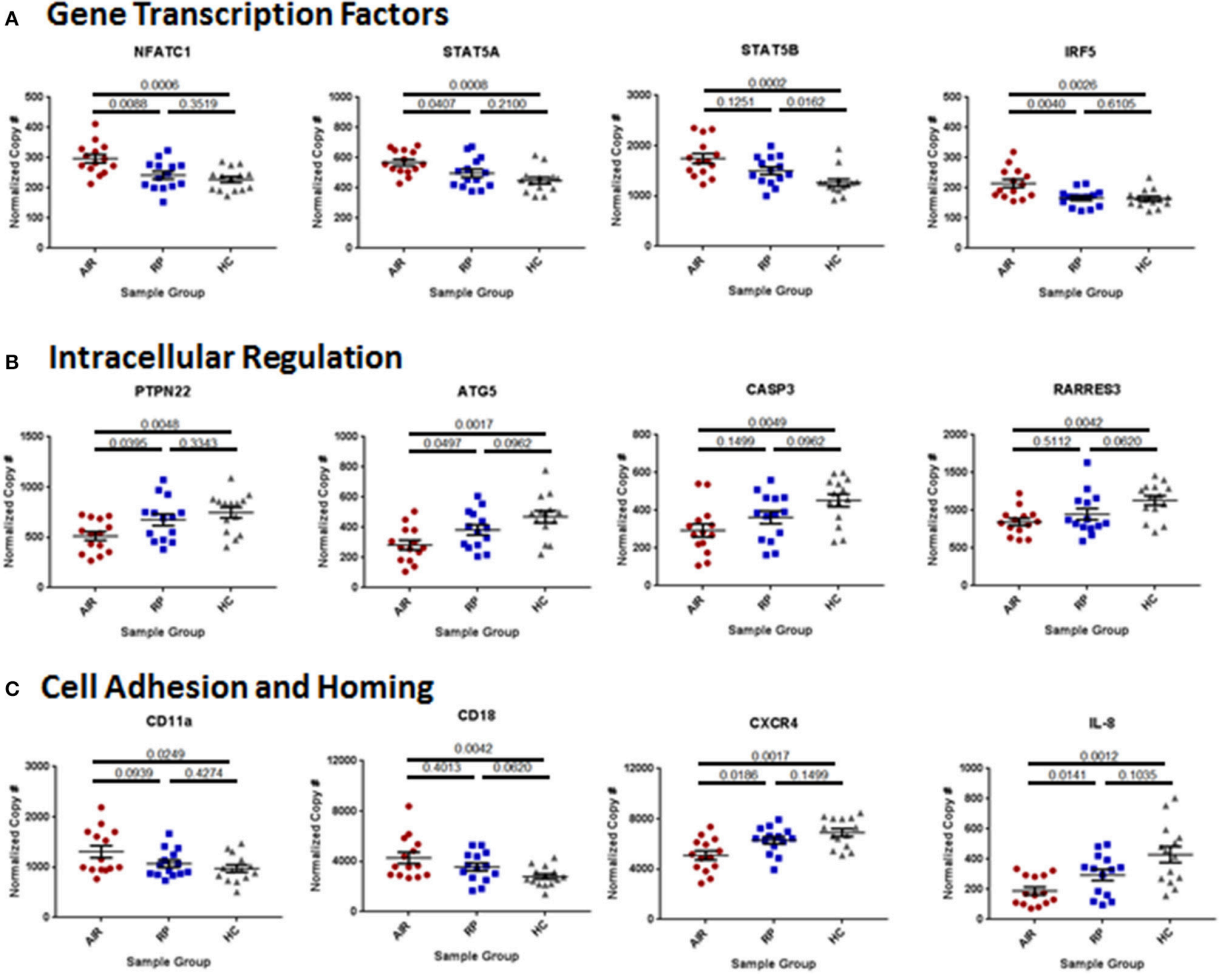
Differences in immune mediator gene expression between AIR patients and control groups. Total RNA was isolated from 2.5 mL of whole blood collected in PAXgene tubes from 14 individuals in each group. Quality of RNA was confirmed in house prior to sending the sample for analysis of mRNA copy numbers using the NanoString Human Immunology Panel. Statistical analysis was performed on the top 445 expressed genes of the 579 immune-associated genes on the panel, of which 53 were very differentially expressed between AIR patients and healthy controls (adjusted *P*-value <0.025). The graphs show normalized and batch-corrected mRNA copy numbers for twelve of the most different genes with each dot representing a different patient. **(A)** Expression of the transcription factors: nuclear factor of activated T cells 1, signal transducer and activator of transcription 5A and 5B, and interferon response factor 5. **(B)** Expression of regulators of cell activation: protein tyrosine phosphatase non-receptor type 22, autophagy related 5, caspase 3, and retinoic acid receptor responder 3. **(C)** Expression of the two subunits (CD11a and CD18) of the cell adhesion molecule: lymphocyte function-associated antigen 1 (LFA-1); expression of CXCR4, the receptor for the chemokine stromal cell-derived factor-1 (SDF-1, CXCL12); and expression of the chemokine interleukin-8 (CXCL8) that is an attractant for neutrophils to sites of infection or inflammation.

Many of the upregulated genes were transcription factors associated with lymphocyte activation particularly downstream of cytokine receptors. These included the nuclear factor of activated T cells 1 (NFATc1), both subunits of the signal transducer and activator of transcription 5 (STAT5), and interferon response factor 5 (IRF5; Figure 5A). Other upregulated transcription factors included IRF3, STAT3, STAT6, B cell CLL/lymphoma 3 (BCL3) and BCL6, and both the P52 (RelB) and P65 (RelA) subunits of nuclear factor kappa B (NFκB; Supplementary Table 2). Among the down regulated genes in the AIR patients, several are involved in suppression of lymphocyte activation and survival and are known to control autoimmunity including: protein tyrosine phosphatase non-receptor 22 (PTPN22), autophagy related 5 (ATG5), caspase 3, and retinoic acid receptor responder 3 (RARRES3; Figure 5B). Cell adhesion and cell homing pathways were also overrepresented among the differentially expressed genes in either direction (Figure 5C and Supplementary Table 2).

## Discussion

This study demonstrates that immune responses against the retinal antigen, recoverin, are more common in humans than previously suspected. PBMC from all of the healthy individuals, AIR patients and RP patients that were tested had measurable IFNγ and IL-10 cytokine release after *in vitro* stimulation with recombinant human recoverin. Median cytokine release for all groups was significantly higher than the spontaneous cytokine production without antigenic stimulation. Anti-recoverin IgG and IgM antibodies were also readily detectable above background in the plasma of almost all individuals, and anti-recoverin IgG1 was significantly higher in a subset of AIR patients than in both control groups. These are the first data that indicate such a high frequency of naturally occurring immunity toward this retinal protein in humans.

The most striking finding in this study was that the levels of IFNγ production by PBMC in response to recoverin was elevated in all of the AIR patients compared to RP and healthy control groups. The amount of IFNγ produced in the assay by AIR patients ranged from 1.25 to 28.44 ng/million cells, while none of the RP patients or healthy controls exceeded the 1.25 ng/million cell level. This difference between AIR patients and the control groups was also evident in the ratio of IFNγ to IL-10 released by PBMC. We believe that this ratio may be the most important determinant because most of the RP patients and healthy controls with higher IFNγ responses toward recoverin also had higher IL-10 levels. Thus, while RP patients and controls averaged ratios of 2.8 and 7.1 fold, respectively, the lowest ratio among the AIR patients was 4.3 fold and only one other patient had a ratio below the average for healthy controls. This data suggests that there is a T_H_1-skewed immune response toward recoverin in the AIR patients with active disease. This change in the balance of IFNγ to IL-10 has been observed in other autoimmune diseases, but has not been previously reported for AIR (20–22). As further evidence of this skew in response, TNFα production in response to recoverin was higher than all control subjects in 5 of 11 AIR patients that were tested (see Supplementary Figure 1B). Notably, we have not been able to detect an appreciable level of IL-17 release from any AIR patient or control PBMC in response to recoverin stimulation using this assay (data not shown). These data indicate a strong induction of a T_H_1 but not a T_H_17 type immune response in patients with untreated AIR.

The nearly equivalent production of IL-10 compared to IFNγ in response to recoverin by PBMC from healthy individuals and RP patients is highly suggestive that recoverin is a natural immune-tolerizing retinal antigen in humans. This finding is in agreement with previous reports that described expression of recoverin and a small number of other retinal antigens in mouse and human thymus, where the formation of natural regulatory T cells (nTreg) is known to occur in response to self-antigen exposure (23, 24). In addition, inducible regulatory T cells (iTreg) may also be generated and supported by expression of recoverin and other retinal antigens by the retina itself (25, 26). Recoverin-specific nTreg or iTreg cells in the retina would be expected to provide local protection from immune attack by secreting IL-10, and possibly expression of other immune regulatory factors in response to presentation of recoverin peptides by local antigen presenting cells (27). This tolerance toward recoverin may be an important contributor to the immune suppressive environment of the eye. Circulating anti-recoverin Treg could also modulate any immune reactions against recoverin occurring outside of the retina, working to maintain the systemic tolerance toward this and potentially other self-antigens. Although the assay system we employed is generally believed to elicit cytokines primarily from T_H_ cells, it is possible that some of the IL-10 released into the culture medium was the product of regulatory B cells or other cellular sources within the PBMC. The recoverin protein used in these assays tested negative for the presence of lipopolysaccharide, which could have elicited IL-10 from other cells in the PBMC if it were present. Further studies are needed to determine the importance of IL-10 in regulating immune tolerance toward recoverin and the relative contributions of different regulatory cell subsets to retinal protection.

The cytokine data highlights the importance of looking for cellular immune responses toward retinal antigens in addition to measuring for ARA. Current laboratory diagnostic testing for AIR relies heavily on finding ARA by single-protein Western blot and/or tissue staining. Although, as shown here, it is clearly possible to detect ARA in AIR patients and there are more specificities found in AIR patients than in RP patients or controls, some major caveats to the utility of the current diagnostic tests exist. Antibodies can have either protective or pathogenic roles in autoimmunity, and their presence is often not changed significantly over time following effective treatment, even with B cell-depletion therapy (28–30). The higher total number and variable specificity of the ARA in AIR patients may be more of an indicator of lost tolerance toward the retina at some point in time, rather than a measure of current disease activity. The current diagnostic techniques also do not provide quantitative data on antibody titers.

The ELISA we developed to detect specific anti-recoverin antibodies was not only positive for circulating IgG and IgM in most of the active AIR patients, but also demonstrated a similar presence of anti-recoverin antibodies in many of the RP patients and individuals with no reported vision loss or significant health conditions. In most of the study participants, these results did not correlate with the presence of a 23 kDal band on the anti-retinal Western blot, suggesting that the whole retina Western blot technique lacked sensitivity and yielded false negative data regarding antibodies specific for recoverin. This may or may not improve if recombinant proteins are used in Western blot, but is not likely to be more sensitive or specific than the ELISA technique. The comparison of ELISA data for IgG1 isotype anti-recoverin antibodies between AIR patients and RP patients did indicate a significant difference in median levels, suggesting that AIR patients may have enhanced IgG1 isotype responsiveness. Elevated titers of IgG1 antibodies specific toward recoverin and other retinal antigens in comparison to other IgG isotypes may prove to be useful biomarkers of disease in some patients. However, it should be noted that circulating IgG antibodies are the product of interactions initiated between activated T_H_ cells and antigen-specific B cells (31–33). Once initiated, antibody production by the resulting differentiated plasma cells is not necessarily T_H_ cell dependent and can continue long after these early interactions have completed. Therefore, if an active T_H_ cell response can be detected in the patient, it is a much stronger sign of current disease activity.

Despite the major differences in T_H_1 immune responsiveness of PBMC toward recoverin in the AIR patients compared to the other groups, there was no evidence of expansion of the T_H_ cell repertoire or other cell types involved in T_H_1 responses (CTL or NK cells) in the peripheral blood of the AIR patients. In fact, there were significantly lower total lymphocyte numbers in both the AIR and RP patient groups compared to healthy controls. There were also lower percentages of circulating CD4^+^ T_H_ cells in the AIR and RP patient groups, which when combined with the lower PBMC counts indicated a very significant deficit of T_H_ cells in the blood of both patient groups. The reasons that both RP patients and AIR patients have fewer T_H_ cells in the blood is currently unclear. The lower number of T_H_ cells appeared to affect all states of activation and was not due to the specific loss of naïve, central memory or effector memory T_H_ cells, but there may have been a more significant loss of the effector memory subset. Thus, the differences in cytokine production in response to recoverin cannot be easily explained by the total numbers of T_H_ cells in the samples. Instead, the higher IFNγ data suggest the presence of a very active subset of recoverin-specific T_H_1 cells within the smaller pool of total T_H_ cells in the blood of AIR patients.

As is the case for most autoimmune diseases, the mechanisms underlying the switch toward T_H_1 cytokine response observed in the AIR patients may involve a combination of genetic and environmental risk factors. Notably, many AIR patients in our clinic have a family or personal history of autoimmunity suggesting that genetic predispositions toward a general loss of immune tolerance may contribute to the risk of AIR. In our study we found that the mRNA levels of the T cell receptor-associated phosphatase PTPN22 were significantly lower in AIR patients than in healthy controls. PTPN22 is a negative regulator of T cell activation that when knocked out in mice leads to significant enhancement of effector memory T cell signaling (34). In contrast, a common gain-of-function polymorphism of PTPN22 has been found in humans that is associated with increased risk in homozygous individuals of developing a wide range of autoimmune diseases (35–37). Thus, PTPN22 appears to act as a critical rheostat on immune cell activation and development of autoimmunity. Genotyping of the patients in this study revealed that none of the AIR patients, RP patients or controls were homozygous for the high risk allele of PTPN22 (data not shown). It remains unknown why the AIR patients had lower PTPN22 mRNA levels or whether this contributed significantly to their disease pathogenesis.

Another difference in mRNA levels that was detected and may be a critical factor in AIR was the decreased expression of major regulator of autophagy, ATG5. Autophagy is important to vision since it is an intracellular process by which retinal cells can detect internal damage and eliminate or recycle cellular debris (38, 39). If there is a deficiency in autophagy, the debris can accumulate and eventually lead to the death of the retinal cell. This is particularly important in retinal pigment epithelial (RPE) cells, since they are intimately involved in response to oxidative stress in the retina and maintenance of photoreceptor health (40). A recent report showed that mice with a deletion of ATG5 or ATG7 specifically in RPE cells developed inflammation in the eye that resulted in an early onset of retinal degeneration (41). Interestingly, ATG7 expression was higher in the AIR patients, suggestive of a compensatory upregulation. It remains to be determined whether increased ATG7 expression may balance out the lower expression of ATG5 systemically or locally, and whether lower ATG5 expression in AIR patients was a specific contributor to or sign of retinal degeneration.

We also detected upregulation of mRNA for a number of genes involved in intracellular signaling and lymphocyte activation. Among these, some of the most significantly different were groupings of genes associated with activated T_H_ cells including: NFATc1, several STAT transcription factors that are associated with cytokine receptor signaling, integrin subunits involved in cell adhesion, interferon response factors, and even the CD4 T helper cell lineage marker. The latter was particularly surprising given the low total number and percentages of circulating CD4^+^ T_H_ cells found in the blood by flow cytometry. These data may signify that the limited number of T_H_ cells that are present in AIR patients are high in transcriptional activation of CD4 but that there may be a defect in translation or post-translational expression. Alternatively, T_H_ cells in another location such as the inflamed eyes of AIR patients may be contributing to the increased T_H_ cell-associated mRNA found in the circulation of these patients.

Immune responses against recoverin have often been reported in the context of CAR but has also been previously found in patients with npAIR (5, 42). It should be noted that none of the patients we evaluated in the current study had evidence of cancer comorbidity at the time of blood draw, nor have any of them been diagnosed with malignancy since then. Many previous studies have shown that anti-recoverin antibodies were associated with cancers in which the malignant cells expressed recoverin, and that there was not much organ-specificity regarding which type of tumors had this capability (43–45). Relatively little data exists showing that human CAR is dependent on induction of T_H_1 immunity toward recoverin, however, one report demonstrated that experimental induction of anti-recoverin CTL in mice led to protection from a recoverin-expressing tumor and simultaneous induction of retinopathy (46). These data suggest that immune-mediated retinal destruction in CAR may be a consequence of the immune system attempting, and possibly succeeding, in the control of tumor progression. An important implication of our findings that healthy individuals have an IL-10-mediated immune response toward recoverin is that expression of recoverin by malignant cells may contribute to enhanced immune regulation in the tumor microenvironment (47). The balance of regulatory versus T_H_1 immunity toward recoverin may have opposing effects on vision and cancer control that will need to be understood as the medical community works toward improved induction of anti-tumor immunity as a major immunotherapeutic treatment strategy against cancer (48, 49). Important steps in addressing this issue may include determining the relative importance of recoverin expression to tumor immune escape, understanding the mechanisms by which cancer cells express recoverin in order to potentially develop methods of specifically blocking recoverin expression by malignant cells, and analyzing recoverin expression by human tumor cells prior to initiation of immunotherapy. The results of these studies may prove beneficial in designing cancer immunotherapy strategies that prevent vision loss in some patients.

## Data and materials availability

NanoString data has been deposited in the GEO archive: Accession #GSE117751. Other data and materials related to this study may be obtained from the authors upon approval of a Material Transfer Agreement from the Regents of the University of Michigan.

## Author contributions

SL designed and directed the research studies. SL, EN, AK, RO, and MO conducted experiments and acquired data. KJ and JH identified and recruited patients and provided clinical information. SL, EN, AK, and RO analyzed the data. RT performed statistical analysis on NanoString data and provided supplementary figures. SL and JH wrote the manuscript.

### Conflict of interest statement

The authors declare that the research was conducted in the absence of any commercial or financial relationships that could be construed as a potential conflict of interest. The reviewer ED and handling Editor declared their shared affiliation.
