# Supplementary material for: T Helper 1 Cellular Immunity Toward Recoverin Is Enhanced in Patients With Active Autoimmune Retinopathy
Source: Front Med (Lausanne). 2018 Sep 13;5:249. doi: 10.3389/fmed.2018.00249 (PMC6146138; doi:10.3389/fmed.2018.00249)
Supplement: Supplementary file 1 [file Data_Sheet_1.pdf]

Supplemental Figure 1

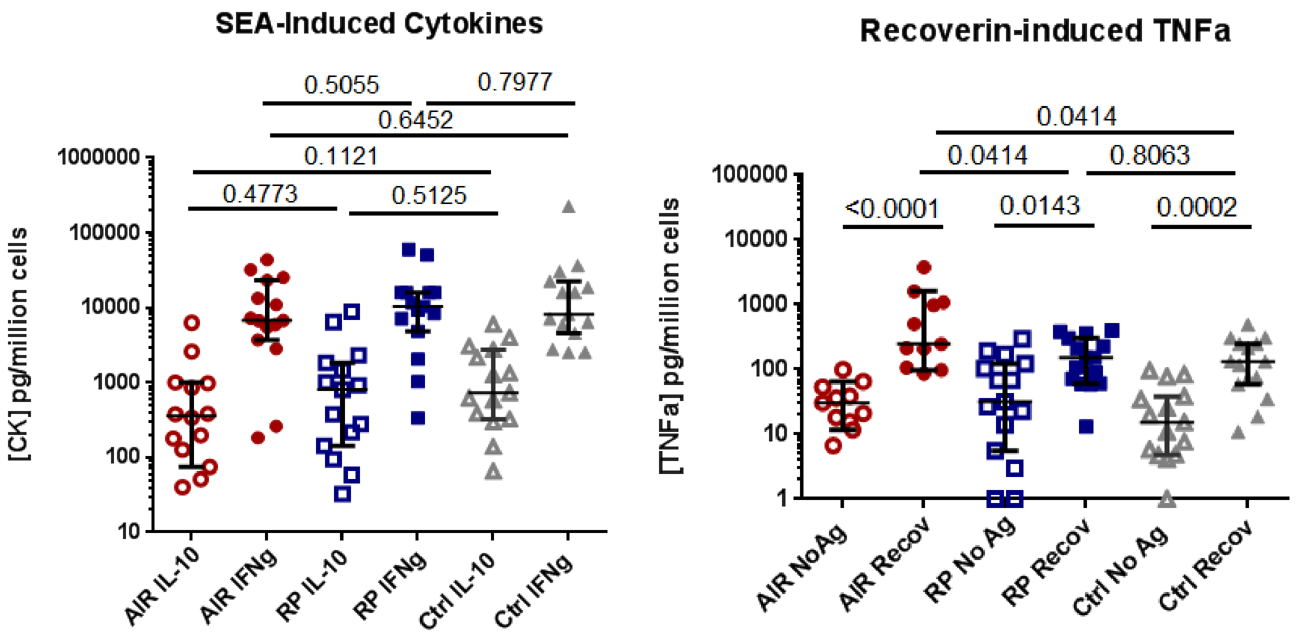

Supplemental Figure 1: PBMC cytokine production in response to the superantigen SEA and TNFa response toward recoverin. A: Isolated PBMC from AIR patients (n=15), RP patients (n=15) and healthy controls (n=14) were cultured for six days in the presence of 10 ng/mL of staphylococcal enterotoxin A. Culture supernatants were collected and the levels of released IFNγ and IL-10 were determined by commercial sandwich ELISA. Each dot represents the mean cytokine level from triplicate wells for each individual. The group median and 95% confidence intervals are shown with horizontal lines and hash marks, respectively. Exact *P* values determined by non-parametric Mann-Whitney tests are expressed below the title of each graph. **B:** Release of tumor necrosis factor alpha in response to recoverin or no antigen was measured by ELISA in cultures of PBMC from AIR patients (n = 11), RP patients (n = 15), and healthy control (n = 14) individuals.

Supplemental Fig. 2

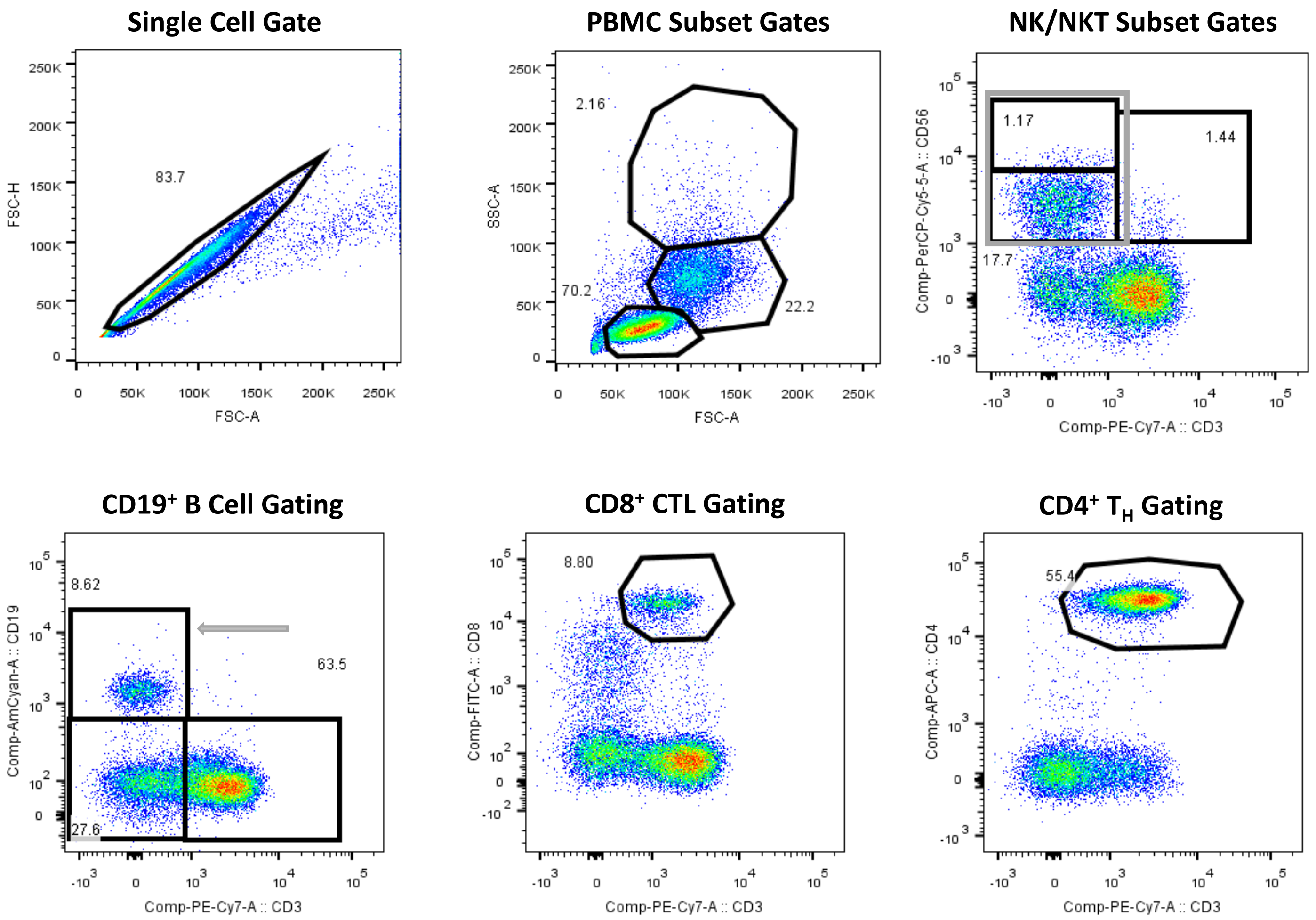

Supplemental Figure 2: Flow cytometric gating for major lymphocyte subsets. The figure shows flow staining data from control subject C010 as representative of gating for all of the samples. Peripheral blood cells were first selected as single cells using forward scatter area (FSC-A) versus forward scatter height (FSC-H) as shown in the upper left dot blot. Next the cells were analyzed for FSC-A versus side scatter area (SSC-A) and gated as small non-granular lymphocytes ($FSC^{low}SSC^{low}$), large low granularity monocytes ($FSC^{high}SSC^{int}$), and large granulocytes ($FSC^{high}SSC^{high}$). Cells in the lymphocyte gate were further analyzed for expression of CD3 as a marker of all T lymphocytes versus the natural killer cell marker CD56 (upper right). NK cell data reported in Figure 4 is the combined percentage of both the $CD3^{neg}CD56^{bright}$ and $CD3^{neg}CD56^{dim}$ populations (grey box), but does not include the $CD3^{+}CD56^{+}$ NKT cell subset. B cells were selected as $CD3^{neg}CD19^{+}$ cells (lower left, grey arrow), and $CD3^{+}$ T cells were subdivided into $CD8^{+}$ cytotoxic T lymphocytes (lower middle), and $CD4^{+}$ T helper cells (lower right).

Supplemental Fig. 3

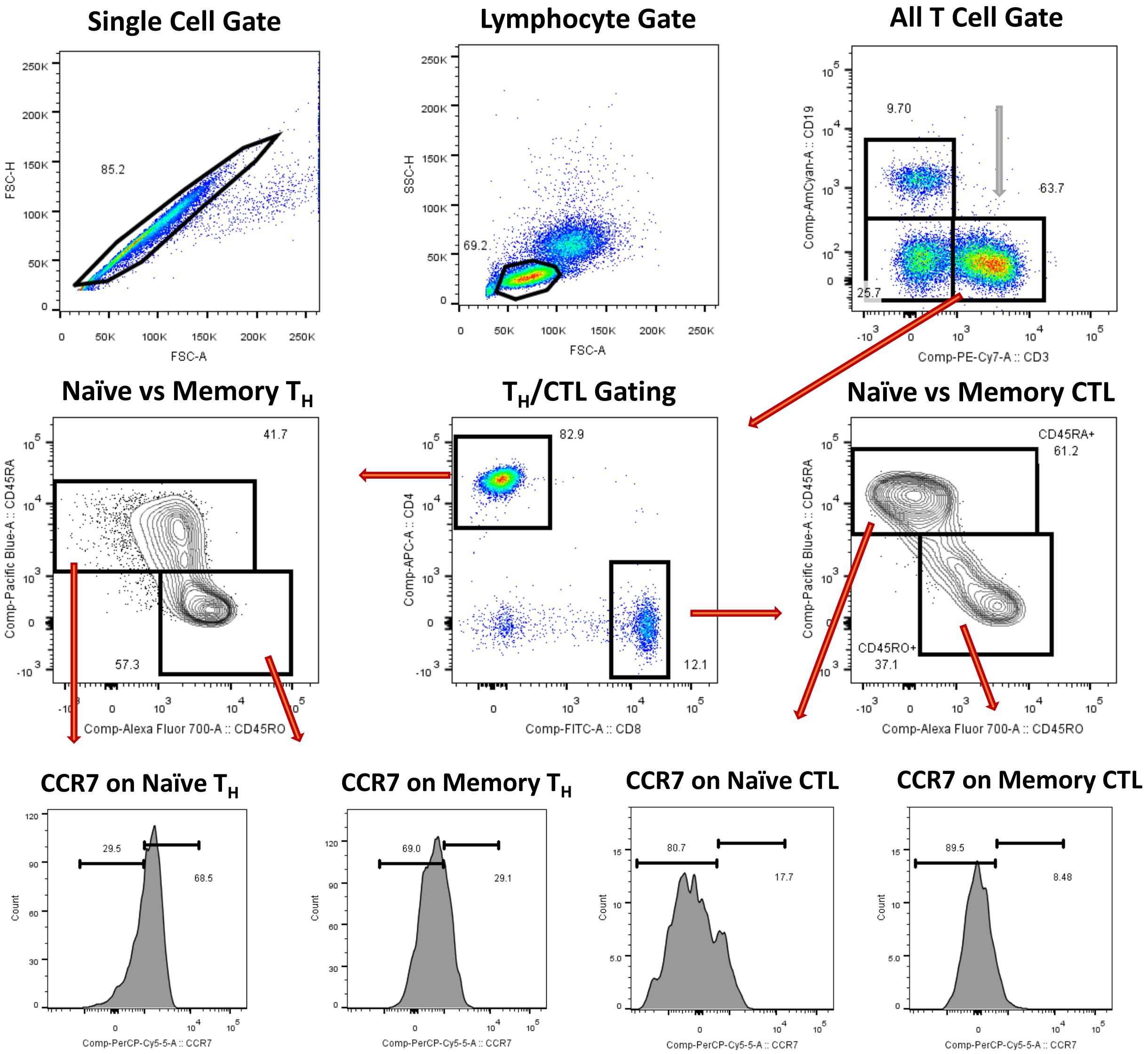

Supplemental Figure 3: Flow cytometric gating for naïve and memory T cell subsets. The figure shows flow staining data from control subject C010 as representative of gating for all of the samples. Peripheral blood cells were first selected as single cells using forward scatter area (FSC-A) versus forward scatter height (FSC-H) as shown in the upper left dot blot. Next the cells were analyzed for FSC-A versus side scatter area (SSC-A) and gated as lymphocytes ($FSC^{low}SSC^{low}$). T lymphocytes were selected for expression of CD3 (upper right grey arrow) and then gated (middle dot blot) into $CD4^{+}$ T helper (T_H) or $CD8^{+}$ cytotoxic T lymphocyte (CTL) subsets. Red arrows indicate the direction for further analysis of the two major T cell subsets into naïve ($CD45RA^{+}$) and memory ($CD45RA^{neg}CD45RO^{+}$) cells. The histograms on the bottom indicate expression of the chemokine receptor CCR7, which separates the naïve and memory T cell subsets into lymphoid organ-homing “central” ($CCR7^{+}$) and non-lymphoid organ-associated “effector” ($CCR7^{neg}$) populations.

Supplemental Fig. 4

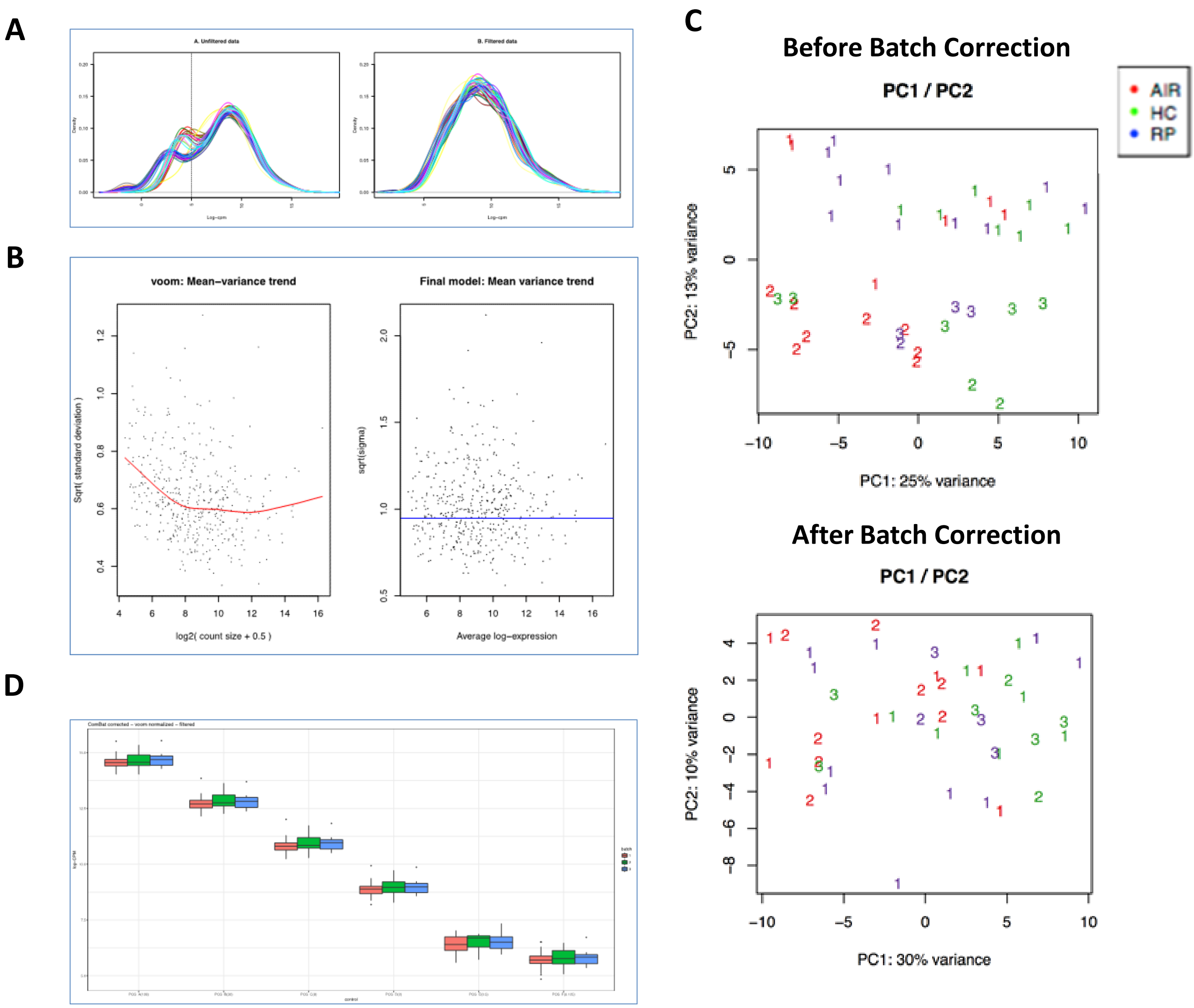

Supplemental Figure 4: NanoString data statistical analysis method. Total RNA was isolated from 2.5mL of whole blood collected in PAXgene tubes from 14 individuals in each group. Quality of RNA was confirmed in house prior to sending the samples for analysis of mRNA copy numbers using the NanoString Human Immunology Panel. Samples were run in several batches over a two year period, and were spiked with a dilution of positive control mRNA at the analysis site to facilitate data normalization and correction of batch to batch variation. All of the sample data was analyzed by a biostatistician. HLA-DQA1 and HLA-DQB1 genes were removed from analysis before processing due to the extremely high variability that could be expected from a heterogeneous human population. **A:** Before and after filtering for low expression. The cutoff of 5 log2 CPM was selected based on comparing the lowest spike-in positive control (Pos_f; 0.125 fM), and the negative controls. In total, 148 genes were removed leaving 445 immune-related genes in the panel. **B:** Raw data from the remaining genes was Voom-transformed to stabilize the mean variance trend, and log(2) transformed. **C:** Batch effects between the three runs (shown as numbers) were corrected using the ComBat algorithm, with the 2016 group ("1") as a reference set. **D:** After batch correction, positive controls were aligned for each batch depicted by the three box plots for each positive control.

Supplemental Fig. 5

A **Control (Housekeeping) Genes**

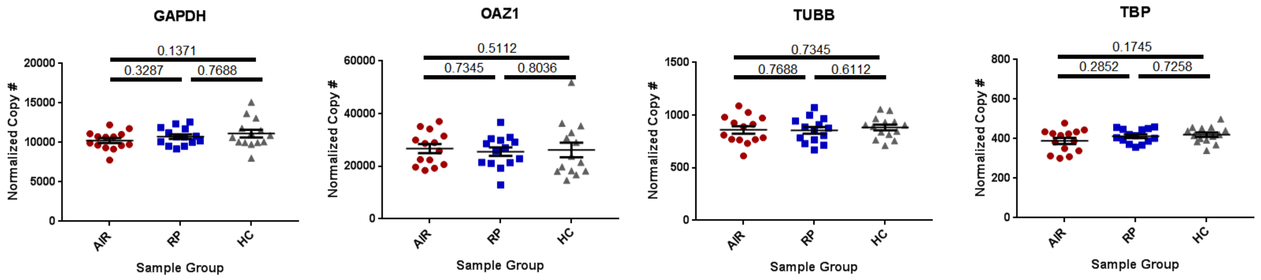

B **Major Lymphocyte Surface Markers**

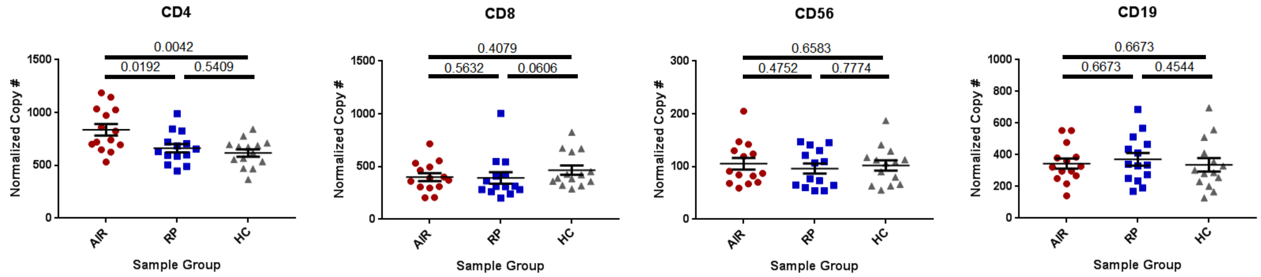

Supplemental Figure 5: NanoString data for selected housekeeping and major lymphocyte surface marker genes. Normalized and batch corrected log(2) NanoString data was reverse-transformed and copy numbers for 445 immune-related genes were determined for AIR patients (n=14), RP patients (n=14) and healthy control subjects (n=14). Each dot represents data for one individual with the group medians and 95% confidence intervals shown with lines and hash marks, respectively. The *P* values determined by Mann-Whitney non-parametric tests for each group comparison are shown below the title of each graph. **A:** Copy numbers of the housekeeping genes glyceraldehyde 3-phosphate dehydrogenase (GAPDH), ornithine decarboxylase antizyme 1 (OAZ1), tubulin beta class I (TUBB), and TATA-box binding protein (TBP) were compared. **B:** Expression of the markers of major lymphocyte lineages: $CD4^+$ T helper (T_H) cells, $CD8^+$ cytotoxic T lymphocytes (CTL), $CD56^+$ natural killer (NK) cells, and $CD19^+$ B lymphocytes were compared.
