# Supplementary material for: T Helper 1 Cellular Immunity Toward Recoverin Is Enhanced in Patients With Active Autoimmune Retinopathy
Source: Front Med (Lausanne). 2018 Sep 13;5:249. doi: 10.3389/fmed.2018.00249 (PMC6146138; doi:10.3389/fmed.2018.00249)
Supplement: Supplementary file 2 [file Data_Sheet_2.docx]

**Supp. Table 1: Subject Demographics and Individual Antibody Data**

| ID | Group | Age | WB #ARA IgG | ~23kD Band^¥^ | No Ag IgM§ | Recov IgM | No Ag IgG | Recov IgG | No Ag IgG1 | Recov IgG1 |
| --- | --- | --- | --- | --- | --- | --- | --- | --- | --- | --- |
| A001 | AIR | 60-65 | 5 | Y | 0.078±0.019 | 0.196±0.019 | 0.108±0.055 | 0.161±0.055 | 0.132±0.028 | 0.172±0.009 |
| A048 | AIR | 70-75 | 3* | Y | 0.117±0.031 | 0.212±0.057 | 0.364±0.012 | 0.340±0.237 | 0.163±0.017 | 0.404±0.022 |
| A052 | AIR | 70-75 | 7 | Y | 0.068±0.012 | 0.385±0.033 | 0.110±0.007 | 0.456±0.012 | 0.087±0.007 | 0.193±0.008 |
| A061 | AIR | 65-70 | 4 | N | 0.061±0.002 | 0.106±0.013 | 0.112±0.007 | 0.260±0.017 | 0.110±0.016 | 0.290±0.036 |
| A070 | AIR | 55-60 | 6 | N | 0.099±0.007 | 0.463±0.020 | 0.138±0.007 | 0.606±0.021 | 0.161±0.004 | 0.198±0.005 |
| A125 | AIR | 70-75 | 9 | Y | 0.078±0.003 | 0.123±0.036 | 0.176±0.030 | 0.451±0.040 | 0.178±0.036 | 0.484±0.025 |
| A132 | AIR | 55-60 | 4 | N | 0.088±0.019 | 0.152±0.017 | 0.111±0.026 | 0.775±0.079 | 0.256±0.081 | 0.733±0.128 |
| A134 | AIR | 50-55 | 5* | N | 0.076±0.004 | 0.350±0.051 | 0.130±0.033 | 0.589±0.071 | 0.372±0.069 | 0.462±0.023 |
| A135 | AIR | 55-60 | 3 | N | 0.077±0.008 | 0.344±0.076 | 0.129±0.042 | 0.677±0.072 | 0.231±0.007 | 0.697±0.046 |
| A145 | AIR | 60-65 | 3 | Y | 0.062±0.004 | 0.078±0.008 | 0.373±0.040 | 0.819±0.053 | 0.126±0.007 | 0.171±0.010 |
| A166 | AIR | 25-30 | 4 | N | 0.109±0.008 | 0.467±0.038 | 0.137±0.037 | 0.650±0.019 | 0.135±0.003 | 0.202±0.022 |
| A178 | AIR | 40-45 | 8* | N | 0.145±0.038 | 0.189±0.048 | 0.319±0.019 | 0.493±0.025 | 0.154±0.013 | 0.300±0.029 |
| A213 | AIR | 55-60 | 5 | Y | 0.102±0.010 | 0.089±0.013 | 0.336±0.077 | 0.308±0.006 | 0.167±0.007 | 0.360±0.021 |
| A277 | AIR | 45-50 | 2* | N | 0.293±0.044 | 0.097±0.045 | 0.080±0.021 | 0.586±0.148 | 0.103±0.002 | 0.160±0.009 |
| A286 | AIR | 40-45 | 9* | Y | 0.056±0.002 | 0.211±0.102 | 0.210±0.089 | 0.739±0.181 | 0.184±0.024 | 0.387±0.005 |
| R152 | RP | 20-25 | 2 | N | 0.064±0.008 | 0.168±0.052 | 0.093±0.003 | 0.557±0.036 | 0.116±0.006 | 0.164±0.009 |
| R172 | RP | 20-25 | 2 | N | 0.053±0.006 | 0.065±0.010 | 0.230±0.028 | 0.256±0.019 | 0.206±0.031 | 0.266±0.026 |
| R188 | RP | 55-60 | 0 | N | 0.145±0.034 | 0.336±0.020 | 0.114±0.011 | 0.425±0.020 | 0.122±0.006 | 0.160±0.006 |
| R201 | RP | 65-70 | 3 | Y | 0.057±0.004 | 0.176±0.093 | 0.075±0.025 | 0.727±0.161 | 0.137±0.017 | 0.187±0.008 |
| R215 | RP | 35-40 | 1 | N | 0.156±0.033 | 0.100±0.021 | 0.211±0.017 | 0.470±0.015 | 0.151±0.018 | 0.292±0.022 |
| R223 | RP | 55-60 | 2 | Y | 0.138±0.016 | 0.137±0.045 | 0.171±0.038 | 0.428±0.031 | 0.138±0.004 | 0.191±0.005 |
| R237 | RP | 55-60 | 2 | N | 0.142±0.026 | 0.275±0.042 | 0.177±0.041 | 0.748±0.066 | 0.070±0.002 | 0.159±0.012 |
| R242 | RP | 60-65 | 3 | N | 0.101±0.023 | 0.101±0.011 | 0.067±0.015 | 0.480±0.096 | 0.062±0.005 | 0.133±0.006 |
| R245 | RP | 30-35 | 0 | N | 0.076±0.011 | 0.127±0.061 | 0.110±0.016 | 0.400±0.032 | 0.100±0.005 | 0.139±0.003 |
| R253 | RP | 45-50 | 0 | N | 0.094±0.033 | 0.140±0.049 | 0.108±0.044 | 0.292±0.075 | 0.106±0.005 | 0.142±0.009 |
| R261 | RP | 75-80 | 1 | N | 0.073±0.018 | 0.080±0.006 | 0.095±0.047 | 0.662±0.036 | 0.067±0.003 | 0.292±0.009 |
| R271 | RP | 35-40 | 3 | N | 0.144±0.059 | 0.101±0.011 | 0.261±0.137 | 0.300±0.164 | 0.169±0.008 | 0.404±0.035 |
| R281 | RP | 35-40 | 2 | N | 0.081±0.037 | 0.536±0.119 | 0.112±0.047 | 0.560±0.056 | 0.362±0.028 | 0.318±0.055 |
| R294 | RP | 70-75 | 2 | N | 0.083±0.032 | 0.396±0.017 | 0.052±0.005 | 0.971±0.177 | 0.093±0.003 | 0.201±0.016 |
| R300 | RP | 35-40 | 3 | Y | 0.144±0.015 | 0.126±0.047 | 0.134±0.053 | 0.248±0.027 | 0.055±0.007 | 0.068±0.001 |
| C010 | HC | 50-55 | 1 | N | 0.054±0.006 | 0.228±0.018 | 0.061±0.018 | 0.500±0.124 | 0.126±0.022 | 0.305±0.021 |
| C230 | HC | 50-55 | 0 | N | 0.084±0.008 | 0.077±0.012 | 0.097±0.017 | 0.143±0.039 | 0.110±0.017 | 0.130±0.011 |
| C249 | HC | 35-40 | 0 | N | 0.066±0.005 | 0.235±0.070 | 0.111±0.064 | 0.422±0.047 | 0.125±0.005 | 0.236±0.033 |
| C272 | HC | 50-55 | 0 | N | 0.059±0.008 | 0.164±0.049 | 0.053±0.007 | 0.704±0.087 | 0.184±0.024 | 0.276±0.009 |
| C273 | HC | 50-55 | 2 | N | 0.070±0.021 | 0.094±0.072 | 0.052±0.006 | 0.586±0.034 | 0.203±0.030 | 0.255±0.031 |
| C275 | HC | 55-60 | 2 | N | 0.054±0.006 | 0.149±0.047 | 0.078±0.002 | 0.503±0.028 | 0.101±0.006 | 0.154±0.012 |
| C319 | HC | 65-70 | 0 | N | 0.097±0.012 | 0.409±0.056 | 0.063±0.015 | 0.555±0.102 | 0.175±0.018 | 0.305±0.040 |
| C323 | HC | 65-70 | 0 | N | 0.078±0.025 | 0.083±0.051 | 0.089±0.037 | 0.373±0.023 | 0.097±0.006 | 0.175±0.012 |
| C324 | HC | 55-60 | 1 | N | 0.097±0.047 | 0.182±0.039 | 0.110±0.059 | 0.330±0.078 | 0.159±0.008 | 0.261±0.010 |
| C360 | HC | 65-70 | 0 | N | 0.117±0.021 | 0.209±0.050 | 0.072±0.012 | 0.417±0.053 | 0.291±0.050 | 0.440±0.059 |
| C361 | HC | 60-65 | 0 | N | 0.064±0.004 | 0.156±0.018 | 0.072±0.006 | 0.393±0.046 | 0.130±0.024 | 0.348±0.015 |
| C362 | HC | 55-60 | 0 | N | 0.082±0.005 | 0.208±0.006 | 0.069±0.005 | 0.348±0.063 | 0.105±0.033 | 0.170±0.035 |
| C363 | HC | 50-55 | 1 | N | 0.056±0.002 | 0.093±0.005 | 0.071±0.003 | 0.454±0.095 | 0.091±0.022 | 0.152±0.009 |
| C364 | HC | 55-60 | 2 | N | 0.078±0.013 | 0.148±0.063 | 0.087±0.009 | 0.231±0.053 | 0.151±0.020 | 0.112±0.002 |

nd = ARA WB not done for this person

*Some Western blots had very faint bands or areas of smeared signals making it difficult to count more bands than listed.

~Recoverin is a 23 kilodalton retinal protein that was not positively detected on every Western blot.

§Numeric values for antibody levels are mean absorbance at 450 nm ± standard deviation of triplicates.

**Supp. Table 2: Most Differentially Expressed Genes (AIR vs Healthy Controls)**


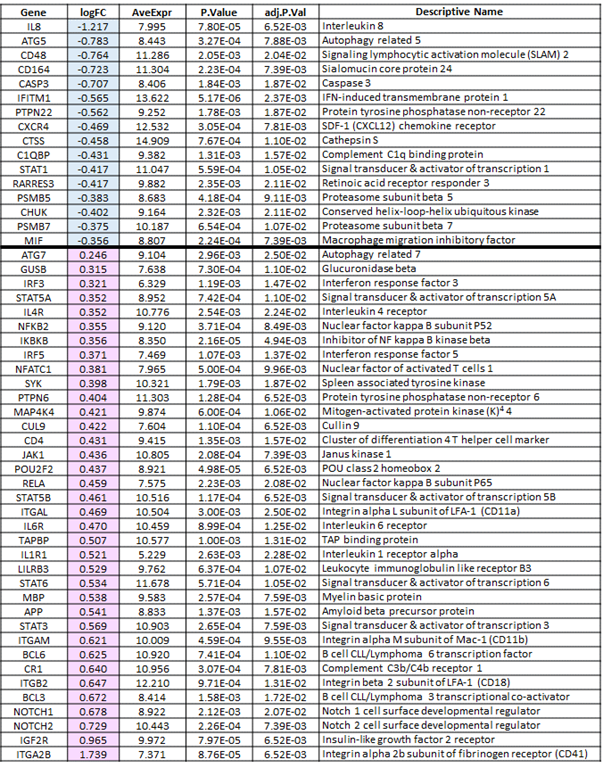


The 16 genes listed at the top (blue logFC column) were lower in AIR patients than in healthy controls.

The 36 genes listed on the bottom (pink logFC column) were higher in AIR patients than in healthy controls.

Average expression values are exponents of log(2) transformation of copy number data for all samples.
